# *Evemphyron sinense*, a new genus and species infesting legume seedpods in China (Coleoptera, Attelabidae, Rhynchitinae)

**DOI:** 10.3897/zookeys.600.6709

**Published:** 2016-06-22

**Authors:** Xiangyang Lv, Miguel A. Alonso-Zarazaga, Zhishu Xiao, Zhiliang Wang, Runzhi Zhang

**Affiliations:** 1 State Key Laboratory of Integrated Management of Pest Insects and Rodents, Institute of Zoology, Chinese Academy of Sciences, Beijing 100101, China; 2 College of Life Science, Hebei University, Baoding, 071002, China; 3 Key Laboratory of Zoological Systematics and Evolution, Institute of Zoology, Chinese Academy of Sciences, Beijing 10010, China; 4 Departamento de Biodiversidad y Biología Evolutiva, Museo Nacional de Ciencias Naturales (CSIC), C/. José Gutiérrez Abascal, 2, E-28006 Madrid, Spain; 5 Museum of Beijing Forestry University, Beijing Forestry University, Beijing 100083, China

**Keywords:** Attelabidae, Callerya dielsiana, Deporaini, east Palaearctic, Eusproda, Fabaceae, legume, new genus, new species, Rhynchitinae, systematics

## Abstract

A new genus *Evemphyron* Alonso-Zarazaga, Lv & Wang, **gen. n.**, belonging to Attelabidae Rhynchitinae, is described. Its single species, *Evemphyron sinense* Alonso-Zarazaga, Lv & Wang, **sp. n.**, was reared from larvae found inside seed pods of the legume *Callerya dielsiana* (Fabaceae, Millettieae) in Sichuan Province (China). The species is figured and placed in the Deporaini because of the presence of minute labial palpi, the strongly crescentic apex of the postmentum, and the apodemes of male IX sternite and female VIII sternite curved sinistro-anterially near their cephalic end. It shows 3-segmented labial palpi and male sex patches on the procoxae, characters that suggest a basal position in the tribe.

## Introduction

**Figures 1–2.**
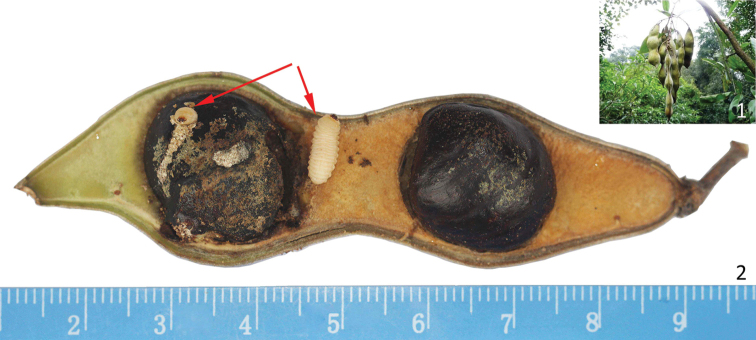
*Callerya dielsiana* **1** Bunch of pods in type locality of *Evemphyron sinense* **2** Opened ripe pod showing seed damage. Red arrows point to two larvae of *Evemphyron sinense*.

As a part of a long-term project on insect-seed interactions, two of the authors (XYL, ZSX) have been investigating the diversity of insect seed predators of woody trees in a subtropical forest of Dujiangyan City (Sichuan Province, China) since 2002. The Dujiangyan region is in the northern part of the Hengduan Mountains, a biodiversity hotspot and priority area for biodiversity conservation in China. Located in the mountains on the western border of the Sichuan Basin, it is in an ecotone between two biogeographical regions, the Qinghai-Tibetan Plateau and the Chengdu Plain. Climatically, it lies in the middle subtropical zone, characterized by evergreen broad-leaved forests. After checking the weevil specimens obtained, a new species belonging to a new genus was identified. It was thought at first sight to belong to the tribe Rhynchitini (Attelabidae: Rhynchitinae). However, an in depth-study of the available material by another author (MAAZ) revealed that the new species belonged in fact to Deporaini. This weevil species was found to infest seedpods of *Callerya dielsiana* (Harms) P.K.Lôc ex Z.Wei & Pedley (Fabaceae) (Figs 1–2). In this study, a detailed description of this new genus and new species is provided, with supporting photographic material.

## Material and methods

On 27 October 2013, 262 weevil larvae were collected from seedpods of the plant *Callerya dielsiana* near Dujiangyan City (Sichuan province). All larvae were placed for adult emergence in a PVC tube (diameter 11 cm, length 40 cm) filled with local soil (30 cm in depth). We checked the emergence of adults once every week from March 2014, and collected adult specimens every day after the emergence of the first adult. In total, nine adult specimens emerged during June, July and September 2014. They were stored in 96% ethanol, and later seven were mounted for the morphological study, leaving two for molecular analysis.

The dry specimens show different degrees of immaturity, mainly affecting their abdomens. Only the male preserved in ethanol was mature enough to allow the extraction of moderately sclerotized genitalia and terminalia. This extraction was done directly in the conserving medium. The abdomen was then soaked overnight in lukewarm 10% sodium hydroxide for digestion of soft tissues. Genitalia and terminalia were photographed in glycerine and later mounted in DMHF (5,5-dimethyl-hydantoin formaldehyde resin) on an acetate card, and pinned together with the tergites and sternites. These have been cross labelled with the specimen in ethanol from which they were extracted.

Descriptions were made using a binocular Nikon SMZ 1500. Photographs (Figures 3–6) were taken with a Canon EOS 700D connected to a Canon MPE-65 lens, Figures 7–8 with a camera attached to a Leica M205 A stereoscopic microscope, Figures 9–10 with a Keyence VHX-1000C Large depth-of-field 3D Digital Microscope, Figures 11–16 with a Canon EOS 5D Mark II mounted on a Nikon SMZ 1500 stereoscopic microscope, Figures 17–19 with an environmental scanning electron microscope FEI Inspect. Extended focus images were generated with Combine ZP 7.0 by Alan Hadley and edited with Adobe Photoshop CS 6.0 if required.

**Figures 3–6.**
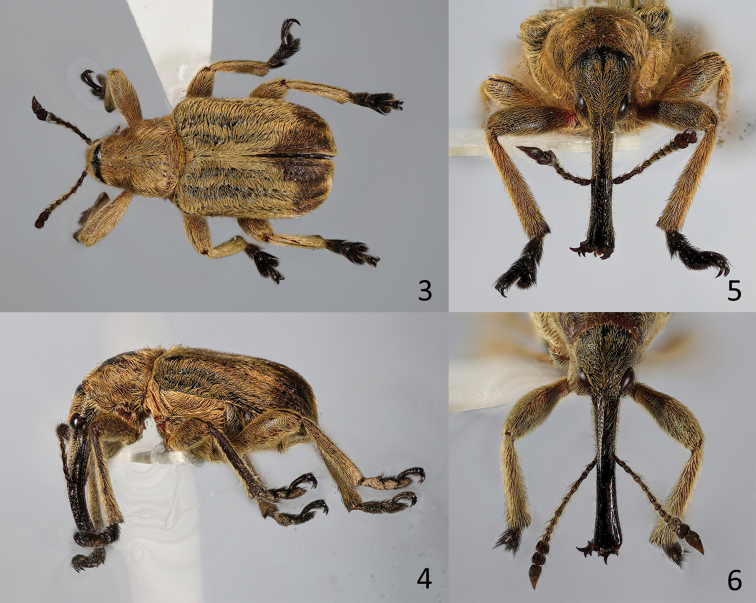
*Evemphyron sinense* **3** Male habitus, paratype, dorsal view **4** Male habitus, paratype, lateral view **5** Male rostrum, paratype, dorsal view **6** Female rostrum, paratype, dorsal view.

**Figures 7–10.**
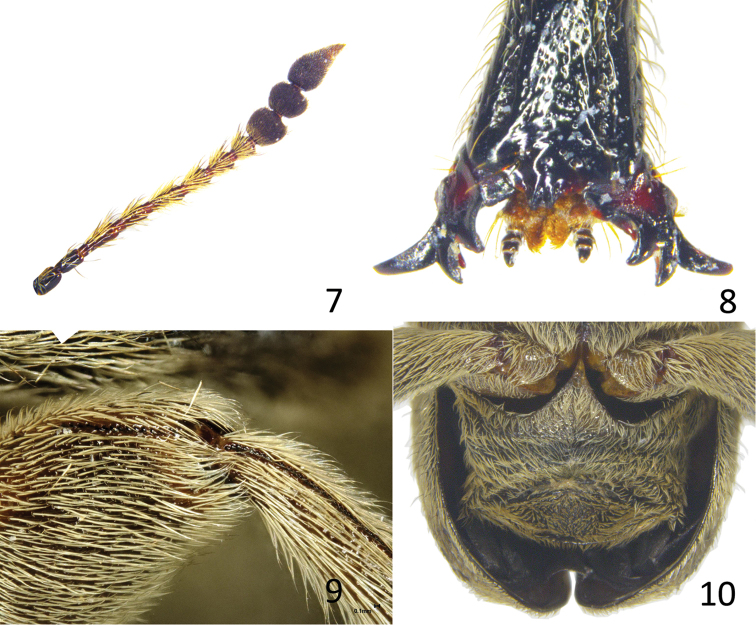
*Evemphyron sinense* **7** Apex of rostrum, paratype, dorsal view **8** Antenna **9** Metafemur and metatibia showing bracteate carina **10** Metacoxae and abdomen, male, ventral view.

Original label data have been written below in Chinese script. Added transliterations into pinyin or translations are placed between square brackets. Data from different labels are separated by two slashes (//) and lines within a label by one slash (/).

Nomenclature follows Alonso-Zarazaga (2011) and in some cases Riedel (2014).

## Taxonomic treatment

### Evemphyron

Taxon classificationAnimaliaColeopteraAttelabidae

Alonso-Zarazaga, Lv & Wang gen. n.

http://zoobank.org/747DBE94-0E6E-4E94-8A24-D0C927515082

Figs 3–6 , 7–10 , 11–16 , 17–19

#### Type species.

*Evemphyron sinense* Alonso-Zarazaga, Lv & Wang, sp. n.

#### Description.

A member of the tribe Deporaini Voss, 1929 as currently understood (cf. Sawada 1993).

*Integument* black to brownish, with green to dark bronze metallic shine, some areas on legs, antennae and underside a little lighter.

*Vestiture* yellow or brown, dense; scales arched to oblique, piliform, those on elytra with apex sometimes flagelliform; brown scales forming a chevron on declivity; scales on dorsum of elytra placed transversally or pointing to outer apical angle of elytra, clearly subparallel to striae only on apical half of 1st interstria, those on anterior half of pronotum, head and metarostrum suberect and pointing forward; scape and funicle with yellow piliform scales, black setae at most as long as scales; tarsomeres densely covered with black piliform scales; very short suberect, arched black setae visible only on apical two thirds of elytral interstriae 9 and 10, rarely visible on other interstriae, and on underside of rostrum.

*Mouthparts*. (Figs 7, 17–18) Mandibles with two teeth on outer margin, a short basal one and a long, sickle-shaped, outwardly-pointed, subapical one, this tooth caducous (in both sexes), leaving at most an obtuse tooth-like remainder after being shed (usually only in females); apex of right mandible with a small ventral cusp (usually quickly worn and obliterated in the apparently more aged specimens, like the outer basal teeth). Maxillary palpi well developed, projecting beyond apex of setose galea, 4-segmented, segments 1-3 transverse, segment 4 subconical, little shorter than wide at base, apex rounded, with eight longitudinal, rod-shaped sensilla. Prementum about as long as wide, asetose, tightly enclosed between deeply crescent-shaped anterior margin of postmentum, with a semicircular base, apex subtruncate, irregular; ligula densely setose, occupying entire apex of prementum; postmental apices almost reaching apices of ligular setae; labial palpi minute, 3-segmented, not protruding from notch in anterolateral corners of prementum and not projecting beyond its apex, first and second palpomeres each with one very long seta, third palpomere minute with two sensilla.

*Rostrum* (Figs 5–6) elongate, in both sexes longer than pronotum, in side view with a strong lower lateral keel running more or less parallel to the ventral margin and a strong median ventral keel parallel to the other, leaving a sulcus between them. Lower margin of scrobe slightly prominent laterally at mesorostrum. Female metarostrum without dense patches of setae.

*Antennae* (Fig. 8) inserted a little behind middle in both sexes, more robust in male; scape shorter than mesorostral width; desmomeres circular in cross-section, 2^nd^ desmomere longer than either scape or first desmomere, but shorter than length of both combined; club loose, slightly flattened, velvety, as long as last 4½ desmomeres, two first segments transverse, last segment longer than any of the others, but as long as or slightly shorter than first and second together, obpyriform, pointed, asymmetrical, its front margin straight to slightly concave, its hind margin convex.

*Head* moderately elongate, subglobose, very weakly constricted behind eyes in side view, but not in dorsal view. Eyes moderately convex, protruding from head outline, in dorsal view longer than minimum distance between them across forehead, in side view slightly oval.

*Pronotum* (Figs 3–4) rather isodiametric, widest in basal quarter, densely punctulate, with rounded, non-carinate sides and base curved towards scutellum, an incomplete median keel present, fine but marked. *Scutellum* (Figs 3–4) subrectangular, slightly transverse, densely punctulate and with dense vestiture.

*Elytra* (Figs 3–4) oblong, dorsal surface flat to evidently concave behind scutellum, with rounded, developed humeri, bases obliquely converging towards scutellum, sides subparallel, falling almost vertically from 7th interstria to costal margin, elytral declivity very steep, elytral apices separately and widely rounded; ten striae formed by rows of strong, more or less rounded punctures, 9th and 10th striae confluent at metacoxal level; scutellar striole absent. Macropterous.

*Ventral areas*. Prosternum short in both sexes, procoxae almost reaching front margin. Hypomera not touching at midline, sternellum large, separating both hypomera and forming part of prothoracic margin (Fig. 19). Procoxae projecting, subconical, tangential to one another. Male with rather large sex patch of setae on inner apex of procoxae. Mesocoxae separated by a distance of less than mesocoxal transverse diameter. Mesocoxal acetabuli open. Abdominal lobes absent, metacoxa reaching metanepisternum (Fig. 10). Tergites I-III fused. Sternites separated by a thin membrane, not visibly fused, sternite 1 barely longer than 2, sternite 5 as long as 4 in both sexes. Propygidium (tergite VI) almost completely covered by elytra, with sparse spicules not forming definite wing-folding patches. Pygidium (tergiteVII) almost vertical, not costate, ca. 1.25 × as wide as long, in dorsal view covered by elytra in both sexes, but clearly visible from behind. Tergite VIII in male without bunches of macrosetae, these irregularly placed along margin.

*Legs*. Femora unarmed. Tibiae straight, moderately flattened, moderately widening towards apex, without mucrones or spurs in both sexes; meso- and metatibiae with an outer crenulate (bracteate) keel (Holloway 1984), this also present but reduced (bracteae scattered) on apical dorsum of meso- and metafemora, represented by a glabrous line (Fig. 9); protibiae with outer margin rounded. First tarsomere subtriangular, little longer than wide in all legs. Claws elongate, inner tooth three quarters as long as outer.

**Figures 11–16.**
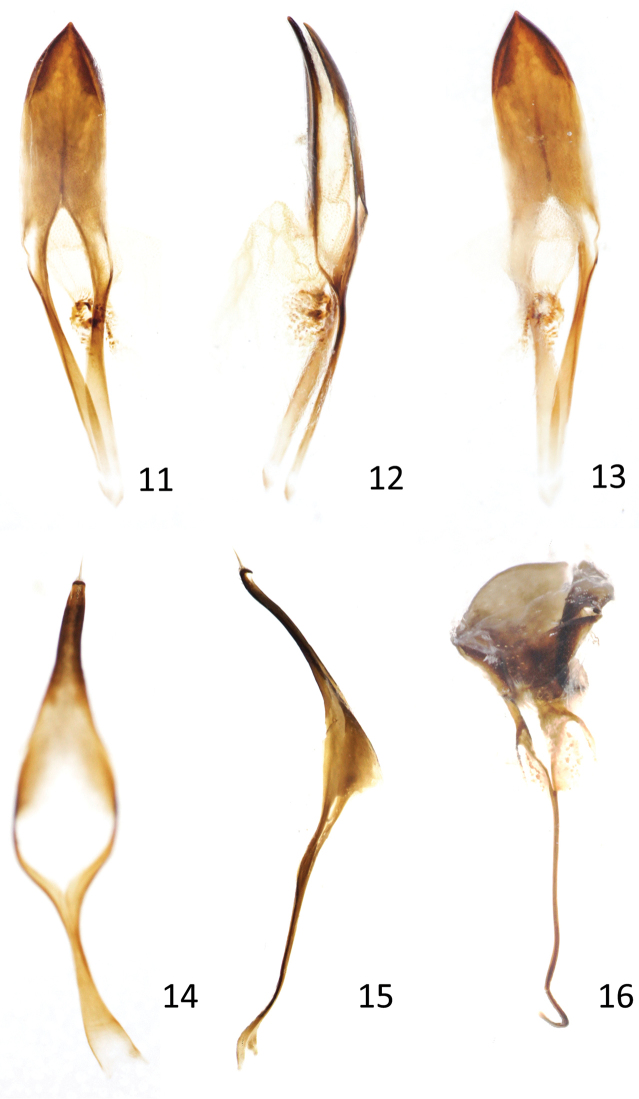
*Evemphyron sinense*, paratype male **11** Penis, dorsal view **12** Penis, lateral view **13** Penis, ventral view **14** Tegmen, dorsal view **15** Tegmen, lateral view **16** Sternite and tergite VIII (to the right) and sternite IX with apodeme turning sinistro-anterially at apex.

*Male genitalia and terminalia*. Penis (Figs 11–13) flattened, in dorsal view pedon apically pointed and mucronulate, tectum almost as wide as pedon, in side view ventral margin of pedon almost straight, weakly incurved at apex, temones moderately widening cephalad. Endophallus wider than tube of penis between the temones, endophallic armature consisting of sparse small denticles, these larger and condensed in an irregularly U-shaped patch between the base of the temones, around the gonopore, with a larger median projection near its anterior margin. Tegmen (Figs 14–15) with dorsal plate strongly projecting and tapering towards apex, this shortly recurved, with a few long and short macrochaetae, manubrium slightly asymmetrical, uniformly broadening to apex. Sternite IX fused to VIII, with apodeme strongly curving sinistro-anteriad near apex (Fig. 16).

**Figures 17–19.**
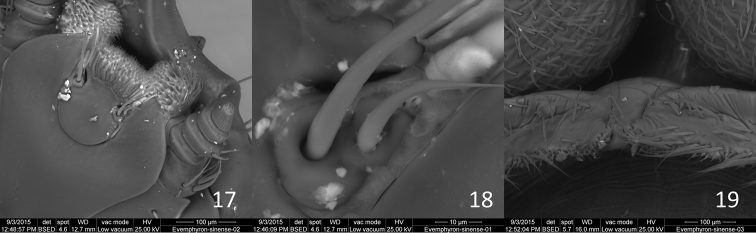
*Evemphyron sinense* **17** Apex of submentum, labium and maxilla **18** Detail of labial palpus **19** Detail of apex of hypomera and intervening sternellum.

*Female genitalia and terminalia*. Ovipositor with gonoxites very wide in anterior half, and obliquely narrowed to an elongate posterior half (“subdivided” in Sawada’s (1993) sense), styli cylindrical, ca. 4 × as long as wide. Spermatheca C-shaped, with cornu robust, apically rounded, a little longer than body, no visible nodulus or ramus, ductus spermathecae and ductus glandulae very close to each other at junction with spermatheca. Sternite VIII with plate slightly longer than wide at base, rounded at apex, manubrium strongly curving sinistro-anteriad near apex.

#### Etymology.

The genus name is based on the classical Greek prefix εὖ (well), latinized as *ev*- (as in *Evacanthus*) and the present active participle of the verb ἐμφύρω (to confuse), ἐμφῦρον (the confusing one). Gender neuter. Stem is *Evemphyront*-.

#### Chinese name.

豆毛象属 [dòu máo xiàng shǔ].

### Evemphyron sinense

Taxon classificationAnimaliaColeopteraAttelabidae

Alonso-Zarazaga, Lv & Wang sp. n.

http://zoobank.org/57EF4590-5C0D-4B43-83F5-C2A7D4317ABD

#### Description.

Characters as given for the genus. In addition:

*Measurements* (in mm) (♂, n=5, ♀, n=2): Body length (standard, without head and rostrum): 6.76–7.33. Rostrum: length: 2.83–3.06 (♂), 3.43–3.46 (♀); width (apical): 0.60–0.67. Distance from antennal insertion to base: 1.13–1.33 (♂), 1.47 (♀). Forehead width: 0.53–0.67. Eye length: 0.67–0.73. Scape: 0.28 × 0.16 (♂), 0.28 × 0.12 (♀). Desmomeres: 1: 0.22 × 0.14 (♂), 0.24 × 0.13 (♀); 2: 0.32 × 0.14 (♂), 0.40 × 0.12 (♀); 3: 0.28 × 0.12 (♂), 0.34 × 0.12 (♀); 4: 0.34 × 0.14 (♂), 0.40 × 0.14 (♀); 5: 0.26 × 0.14 (♂), 0.24 × 0.14 (♀); 6: 0.24 × 0.14 (♂), 0.22 × 0.14 (♀); 7: 0.18 × 0.18 (♂), 0.20 × 0.16 (♀). Club: 1: 0.30 × 0.32 (♂), 0.30 × 0.30 (♀); 2: 0.28 × 0.34 (♂,♀); 3: 0.54 × 0.30 (♂), 0.58 × 0.28 (♀). Pronotum: length: 2.33–2.43 (♂), 2.26–2.30 (♀); maximum width: 2.26–2.37. Elytra: length: 4.43–4.90; maximum width: 3.30–3.46.

*Rostrum* 1.17–1.27 × as long as pronotum in male (Fig. 5), 1.50–1.52 in female (Fig. 6), in dorsal view narrowest slightly behind antennal insertion, prorostrum widening towards apex, metarostrum towards base, apex rounded, medially with bidentate projection, prorostrum with one densely punctate lateral sulcus on each side and with dorsum densely punctulate in apical half, the punctures becoming sparser and larger behind, metarostrum with median, wide, impunctate and glabrous keel and two low lateral keels hidden under the dense, semierect, pointing forward scales; in side view, rostrum moderately curved, uniting with head at mid height of the latter, prorostrum tapering to apex; in ventral view with lateroventral keels marking the ventral borders of rostrum, and one low median keel having on each side a low sulcus with oblong punctures, these moderately setose.

*Antennae* inserted at 0.39–0.44 from base of rostrum in male, at 0.42–0.43 in female, articles with integument shining, with moderately long scales and setae, except velvety club, only with a few setae; scape and pedicel oblong, other desmomeres subcylindrical, except 7^th^, subglobular to suboblong.

*Head* with forehead forming a very obtuse to almost flat angle with rostrum in side view, forehead densely punctate and pubescent, scales pointing forward, underside of head with strong transverse rugae, these prominent in side view.

*Pronotum* slightly depressed transversely behind front margin and before hind one, the punctures in the depressions more confused than in the remaining surface.

*Elytra* 1.33–1.43 × as long as wide, with interstriae densely punctulate, punctures ca. 1/6 the diameter of those forming the striae. Size of strial punctures decreasing towards apex of elytra.

*Legs* similar in both sexes, devoid of any sexual character.

*Wings* blackish.

#### Material examined.

Nine specimens, rather teneral. All printed labels with 中国科学院动物研究所 [Zhōngguó kēxuéyuàn dòngwù yánjiū suǒ, Chinese Academy of Sciences, Institute of Zoology]. All specimens are deposited in this institution, except one male and one female paratype, which are deposited in the Alonso-Zarazaga collection (Museo Nacional de Ciencias Naturales, Madrid, Spain).

Holotype: 1 male, labelled: printed: 2014-VII-31 / 四川 都江堰 浦阳镇 花 / 溪村 [Sìchuān, Dūjiāngyàn, Pŭyángzhèn, Huāxīcūn], 肖治术 [Xiāo Zhìshù] leg. // printed: N31°03'45.07" / E103°43'0.52" / Alt. 709 m // printed: 寄主 香花鸡血藤 [jìzhǔ [host] xiānghuā jī xuè téng] / *Callerya dielsiana* (Harms) // handwritten: A037 / 1(in red) ♂ / H.7.31.

Paratypes: 1 male, same data as holotype, except dated 2014-VII-12 and a handwritten label: A037, 14-7-12 / 山胡豆[Shānhúdòu], 左后[zuǒhòu] / 足断[zúduàn] ♂; 1 male, same data as holotype, except dated 2014-VII-7 and a handwritten label: A037 / 1(in red) ♂ H.7.7 (specimen had been dissected, the abdomen had been discarded, some pieces in a glycerine vial); 1 male, same data as holotype, except dated 2014-VI-24, and a handwritten label: A037 / H / ♂ 6.24; 1 male, same data as holotype, except dated 2014-VII-7, and a handwritten label: A037 / 2 (in red) / ♂ H.7.7.; 1 female, same data as holotype, except dated 2014-VI-20, and a handwritten label: A037 / ♀ H.6.20; 1 female, same data as holotype, with a handwritten label: A037 / 2 (in red) / ♀ H.7.31 (specimen dissected, abdomen discarded, some pieces in a glycerine vial); 2 males, same data as holotype, but dated 2014-VI-12 and 2014-IX-17 respectively, conserved in pure ethanol vials for DNA extraction. (one of them, being less teneral, has been dissected for the study of the male genitalia and terminalia).

#### Etymology.

The species is named after the country where it has been found, China (in Latin: *sinensis, -e*: Chinese). It is an adjective, in neuter form to agree with the gender of the genus.

#### Chinese name.

中华豆毛象 [zhōnghuá dòu máo xiàng].

#### Host plant.

*Callerya dielsiana* (Harms) P.K.Lôc ex Z.Wei & Pedley (Fabaceae, Millettieae). Larvae develop in seeds inside the pods. This weevil species was not found on other plant species despite long-term collecting of several Fabaceae species and other possible host plants carried out at the same study site.

#### Distribution.

This species is known only from the type locality in Sichuan Province (China).

## Discussion

This genus is superficially similar to *Cyllorhynchites* Voss, 1930, mainly in the head not being constricted behind the eyes and the presence of dense yellowish piliform vestiture, somewhat reminiscent of that of Cyllorhynchites (Cyllorhynchites) ursulus rostralis (Voss, 1930), a common weevil in China. This led to the initial placement in the tribe “Rhynchitini”, *sensu* Riedel (2014). However, the characters of the minute labial palpi and the strongly crescentic apex of the postmentum are quite uncommon in that tribe, and more similar to the state found in members of the Deporaini (cf. Sawada 1987, 1988, 1993). Study of the male and female genitalia supported the latter placement, since the apodeme of male sternite IX and female sternite VIII curve sinistro-anteriad in both the new genus and the Deporaini. Thus Sawada’s (1988, 1993) characters defining the tribe Deporaini are partly met, even if the propygidium is hardly visible, the head is not constricted in dorsal view between vertex and occiput, and the labial palpi are 3-segmented.

The relationships of *Evemphyron* within Deporaini are also contentious. In fact, no genus known to belong to this tribe seems to be closely related. The keys provided by Legalov (2007) are confusing and do not help to locate a close genus, specimens of the new genus being taken to such disparate taxa as *Depasophilus* Voss, 1922, *Pseudocoenorrhinus* Voss, 1935, *Pseudodeporaus* Voss, 1922 or *Caenorhinus* s.l., depending upon which of the conflicting characters is given more strength.

The new genus should belong, according to the keys proposed by Legalov (2003, 2007) to the subtribe Depasophilina Legalov, 2003, defined by its author on a single character: the covered propygidium. The appreciation of this character is rather subjective, the placement of the latter depending on the maturity of the specimen and the way the specimen is prepared (pinned, glued, etc.). Legalov’s (2003) description of this subtribe can be applied to genera of the tribe Rhynchitini as well. No mention is made of the genitalic structures. Depasophilina are reddish, thinly pubescent, tropical animals of unknown habits. A close relationship of the new genus with them seems improbable.

If the character of the covered propygidium is not taken into consideration, the only possible placement is within the subtribe Deporaina. However, this is also a very disparate group regarding its contents. The combination of 3-segmented labial palpi, propygidium practically covered by the elytra and lacking definite wing folding patches, tibiae neither spurred nor mucronate in both sexes, long rostrum without long, dense patches of long hairs in female and male procoxae with sex patch does not indicate any included genus as an obvious close relative. This set of character states suggests that the new genus could be very primitive (or even the most primitive) in the subtribe, since the palpi segment number, the long rostrum and the male sex patch on the procoxae are clearly symplesiomorphies shared with genera of Byctiscini, Rhynchitini and Auletini, but they are absent from advanced members of Deporaini.

*Eusproda* shares with *Evemphyron* the male sex patch on the procoxae (a symplesiomorphy suggesting the basal position of both genera) and the trophic link to Fabaceae, although *Eusproda* behaves as a shoot cutter of kudzu (*Pueraria montana* (Lour.) Merr.) and Japanese clover (*Lespedeza cyrtobotrya* Miq.) (Fabaceae, Phaseoleae and Desmodieae, respectively), and not as an ovary or young fruit driller, as is the case for *Evemphyron sinense*. They also share the long rostrum, the very weak basal constriction of the head, the antennae inserted just behind middle of the rostrum, the non-contiguous hypomera, the subisodiametric pronotum, the absence of a scutellar striole, the tibiae without mucrones or spurs, overall similarity of the male genitalia and the ovipositor divided into two regions and with styli. Table 1 summarizes the differences between both genera.

**Table 1.** Character comparison between *Eusproda* and *Evemphyron* gen. n.

Character	*Eusproda*	*Evemphyron*
Integument	black with blue metallic shine mostly on elytra	black to brownish, with green to dark bronze metallic shine, some areas of appendages and abdomen lighter
Vestiture	sparse, thin, brown	dense, comprised of yellow and brown piliform scales
Base of rostrum in female	with dense long hairs	without long hairs
Labial palpi	2-segmented	3-segmented
Second desmomere	about as long as scape or 1^st^ desmomere	longer than scape or 1^st^ desmomere
Last antennal club segment	as long as 1^st^ or 2^nd^, symmetrical	longer than 1^st^ or 2^nd^, asymmetrical
Eye length in dorsal view	less than forehead width	more than forehead width
Pronotum	without median keel	with shortened median keel
Scutellum	oblong	slightly transverse
Elytra	elongate, ca. 1.7 × as long as wide	shorter, 1.33-1.43 × as long as wide
Elytra	uniformly convex	dorsally flat to concave behind scutellum
Elytral striae 9^th^ and 10^th^	confluent near apex of elytra	confluent at metacoxal level
Propygidium	with wing folding patches	without wing folding patches
Metatarsomere 1	slightly longer than 2+3	clearly shorter than 2+3
Tegminal arms	broad, angulate	thin, curved
Tegminal manubrium	strongly asymmetrical, T-shaped at apex	slightly asymmetrical, uniformly broadened
Size	smaller (3.5-4.5 mm, without rostrum)	larger (6.76-7.33, without head and rostrum)
Biology	shoot-cutter	ovary- and young-fruit- driller

*Evemphyron* could be close to any of the genera related to *Deporaus* Samouelle, 1819. However, the same combination of characters precludes the finding of another genus sharing putative synapomorphic features, as no known genus matches the combination found in *Evemphyron*. *Caenorhinus* C.G. Thomson, 1859 shares with *Evemphyron* the confluence of 9^th^ and 10^th^ elytral stria at metacoxal level and the absence of wing folding patches on the propygidium, but differs clearly by the presence of spurs in at least one pair of tibiae in both sexes and of mucrones at least in one pair of tibiae in males, the shorter, apically widened rostrum and the presence of defined endophallic sclerites.

In summary, this new genus is placed in Deporaini Deporaina on the basis of the minute labial palpi, the strongly crescentic apex of postmentum, the absence of scutellar striole, and the apodemes of male sternite IX and female sternite VIII curved sinistro-anteriad near their cephalic end.

The definitive placement of this new genus will have to wait until a molecular phylogeny of the tribe (and the subfamily Rhynchitinae as a whole) is performed.

## Supplementary Material

XML Treatment for Evemphyron

XML Treatment for Evemphyron sinense
